# Integration of *Aspergillus niger* transcriptomic profile with metabolic model identifies potential targets to optimise citric acid production from lignocellulosic hydrolysate

**DOI:** 10.1186/s13068-021-02099-2

**Published:** 2022-01-12

**Authors:** Daniel J. Upton, Mehak Kaushal, Caragh Whitehead, Laura Faas, Leonardo D. Gomez, Simon J. McQueen-Mason, Shireesh Srivastava, A. Jamie Wood

**Affiliations:** 1 Department of Biology, University of York, Wentworth Way, York, YO10 5DD UK; 2 Systems Biology for Biofuel Group, International Centre for Genetic Engineering and Biotechnology (ICGEB), ICGEB Campus, Aruna Asaf Ali Marg, New Delhi, 110067 India; 3 Department of Mathematics, University of York, Heslington, York, YO10 5DD UK

**Keywords:** Fermentation, Citric acid, *Aspergillus niger*, Metabolic modelling, Transcriptomics

## Abstract

**Background:**

Citric acid is typically produced industrially by *Aspergillus niger*-mediated fermentation of a sucrose-based feedstock, such as molasses. The fungus *Aspergillus niger* has the potential to utilise lignocellulosic biomass, such as bagasse, for industrial-scale citric acid production, but realising this potential requires strain optimisation. Systems biology can accelerate strain engineering by systematic target identification, facilitated by methods for the integration of omics data into a high-quality metabolic model. In this work, we perform transcriptomic analysis to determine the temporal expression changes during fermentation of bagasse hydrolysate and develop an evolutionary algorithm to integrate the transcriptomic data with the available metabolic model to identify potential targets for strain engineering.

**Results:**

The novel integrated procedure matures our understanding of suboptimal citric acid production and reveals potential targets for strain engineering, including targets consistent with the literature such as the up-regulation of citrate export and pyruvate carboxylase as well as novel targets such as the down-regulation of inorganic diphosphatase.

**Conclusions:**

In this study, we demonstrate the production of citric acid from lignocellulosic hydrolysate and show how transcriptomic data across multiple timepoints can be coupled with evolutionary and metabolic modelling to identify potential targets for further engineering to maximise productivity from a chosen feedstock. The in silico strategies employed in this study can be applied to other biotechnological goals, assisting efforts to harness the potential of microorganisms for bio-based production of valuable chemicals.

**Supplementary Information:**

The online version contains supplementary material available at 10.1186/s13068-021-02099-2.

## Background

For a century, the filamentous fungus *Aspergillus niger* has been used industrially for the production of citric acid; currently, production exceeds 2 million tonnes a year [1]. The ease of culture and its tolerance to typical industrial fermentation stresses make *A. niger* [2] a desirable organism for industrial applications. Beyond its established uses, *A. niger* also has potential to produce other valuable chemicals including succinic [3] and itaconic acid [4].

The commercial production of citric acid by *A. niger* fermentation is dependent on sucrose-based feedstocks, primarily molasses [5]. In this regard, *A. niger* is underexploited as it is saprophytic in nature with an ability to assimilate at least 69 carbon sources and 30 nitrogen sources [6]. There is an increasing need to unlock this metabolic potential, so that *A. niger* can play a key role in harnessing the value of underutilised second-generation feedstocks for the bioeconomy [7]. One such feedstock is sugarcane bagasse, the main by-product of sugarcane processing and a potential source of lignocellulosic sugars. Global sugarcane production was around 1900 million tonnes in 2013 [8], generating around half a billion tonnes of bagasse. To achieve cost-competitive citric acid production from bagasse hydrolysate requires the optimisation of strains away from sucrose-based fermentation to bagasse hydrolysate as the fermentation medium.

Strain optimisation can be achieved either via cycles of random mutagenesis and selection or by targeted engineering. The former is well demonstrated for citric acid production by *A. niger* [9], and although successful, its iterative nature makes it laborious and requires a suitable selection and evolution strategy to be available or designed. Rational strain engineering provides a faster strain development process that achieves the required genetic changes in a more stable manner. Optimising strains via targeted engineering is dependent on a metabolic understanding of the target organism and an ability to accurately identify targets. The establishment of omics technologies has enabled researchers to develop a more comprehensive understanding of the target organism; however, this can be challenging given the volume of data from omics analyses. One core systems biology method, constraint-based metabolic modelling, has now developed an extraordinary number of differing methods to address this challenge and integrate omics data with metabolic models [10–16].

In this study, we highlight the potential of bagasse as a feedstock for citric acid production, examining the performance of *A. niger* for the fermentation of bagasse hydrolysate to citric acid. Using fermentative time series data, we adapted our dynamic model [17] to capture the dynamics of bagasse hydrolysate fermentation. We show that the performance of the strain in this study is suboptimal and investigate further using transcriptome analysis at key fermentation timepoints. By employing a novel method involving an evolutionary algorithm guided by transcriptome data, we identify targets to achieve optimal citric acid productivity from bagasse hydrolysate.

## Results

### Fermenting sugarcane bagasse hydrolysate to produce citric acid

**Fig. 1:**
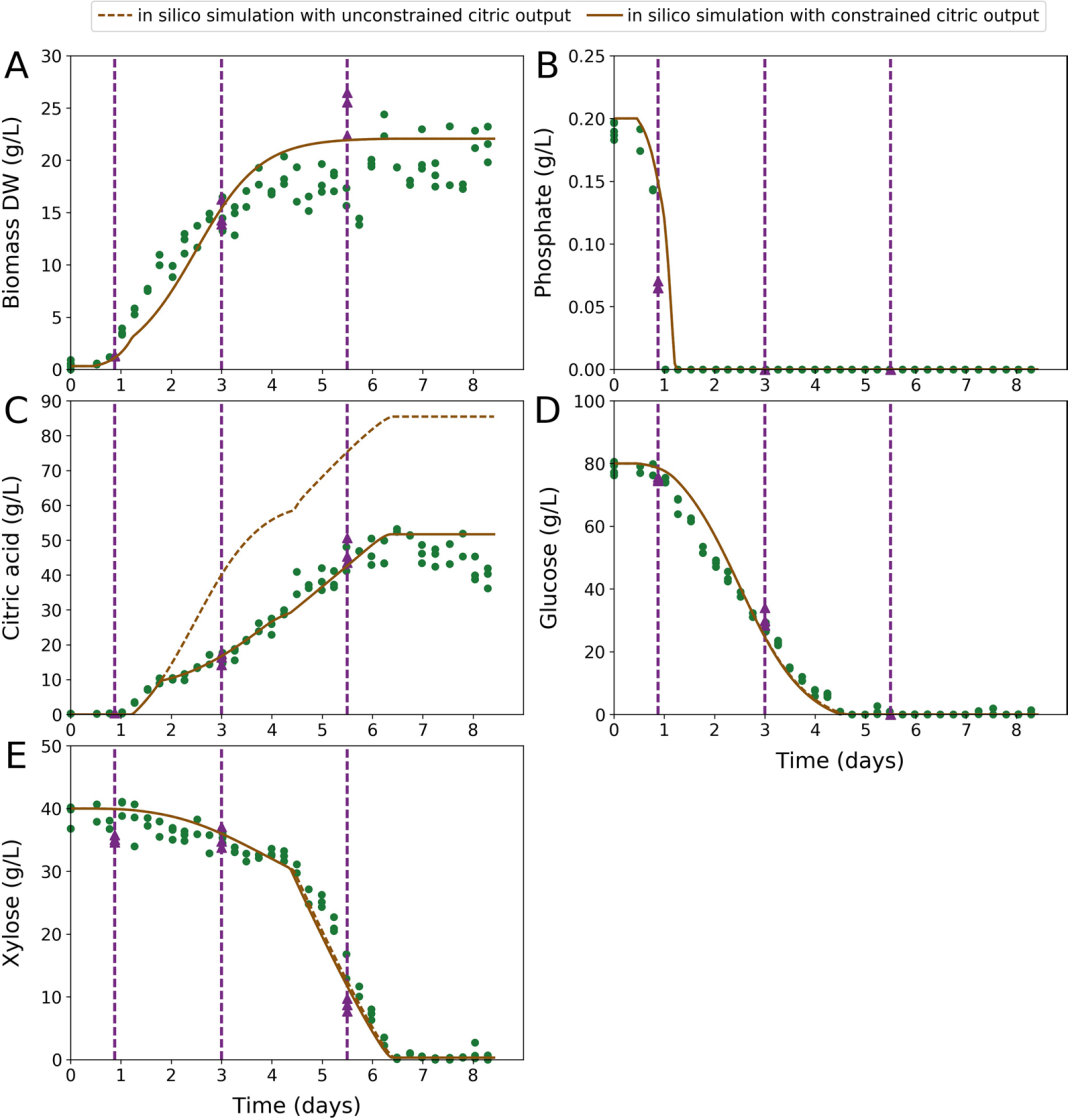
Time series of sugarcane bagasse hydrolysate fermentation with dynamic modelling. Green dots correspond to in vivo fermentation data. Purple dashed vertical lines indicate timepoints chosen for transcriptome analysis. Purple triangles correspond to data from cultures used for transcriptome sampling. Solid brown lines represent in silico data from a simulation with citric output constrained to fit the in vivo data. **A** Change in biomass dry weight (g/L) over time. **B** Change in external phosphate concentration (g/L) over time. **C** Change in external citric acid concentration (g/L) over time. Dashed brown line represents in silico data from a simulation with unconstrained citric output. **D** Change in external glucose concentration (g/L) over time. **E** Change in external xylose concentration (g/L) over time. Individual data-points are shown

To evaluate the fermentation of sugarcane bagasse hydrolysate for the production of citric acid, we obtained fermentative time series data on citric and biomass output as well as glucose, xylose, and phosphate input. From a hydrolysate containing 120 g/L total sugars consisting of glucose (80 g/L) and xylose (40 g/L), 50 g/L citric acid was produced in 6 days (Fig. 1). Glucose was fully consumed by day 5 at which point xylose consumption increased significantly with full consumption of sugars by day 7, indicating a sequential uptake mechanism. We observed similar characteristics to citric acid fermentations performed previously [17] with the onset of citric acid production coinciding with the full depletion of external phosphate and a switch to phosphate-limited growth.

### Simulating the fermentation of sugarcane bagasse hydrolysate to citric acid by dynamic modelling

To capture the dynamics of sugarcane bagasse hydrolysate fermentation in silico, we adapted our dynamic modelling framework [17] to reflect mixed glucose/xylose fermentations. The adapted model simulates the sequential uptake of glucose and xylose and with adjustments made to kinetic parameters (see Methods) gives close fits to the in vivo fermentation data (Fig. 1). The model estimated that citric acid titres could reach a maximum of 85 g/L, almost twofold higher than what we observed in vivo (Fig. 1). By imposing a constraint on citric acid output in silico, the model was able to reflect in vivo citric acid production (Fig. 1), suggesting the strain we used is suboptimal and highlighting the need for strain optimisation to realise optimal productivity.

### Transcriptomic analysis at selected timepoints to investigate the fermentation of sugarcane bagasse hydrolysate to citric acid

**Fig. 2:**
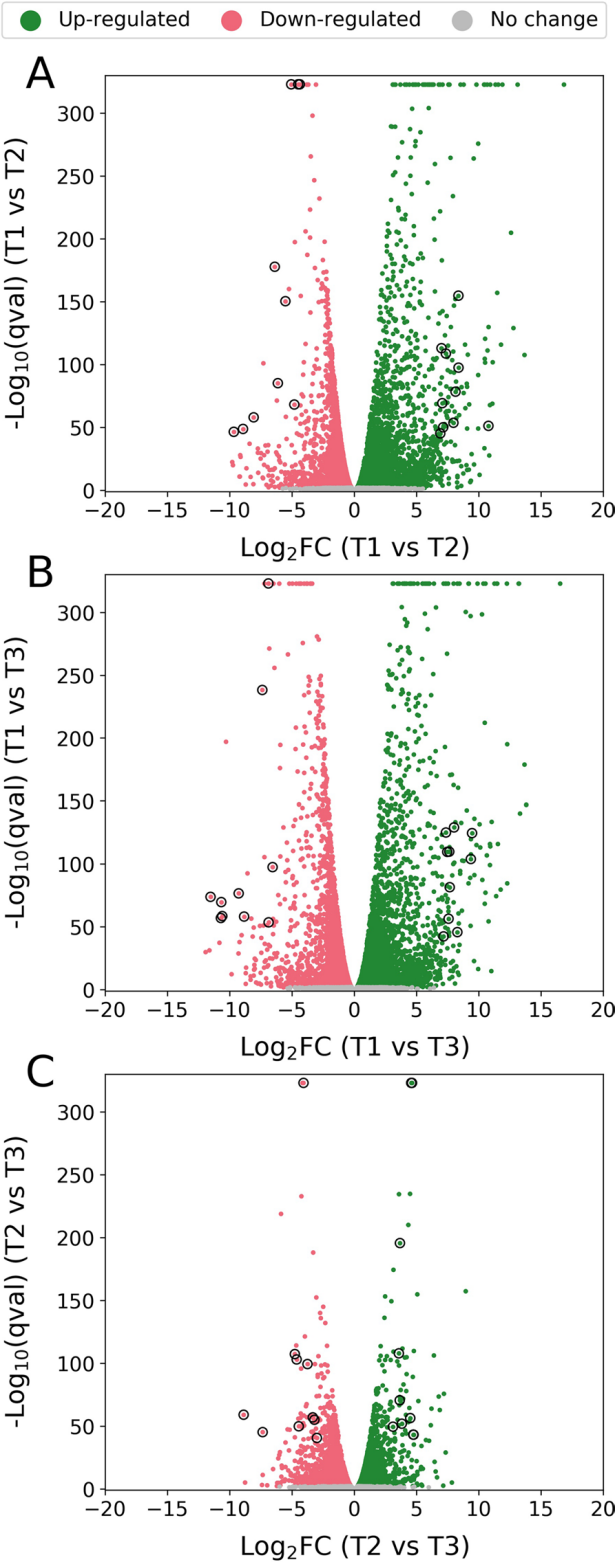
Volcano plots showing the differential expression between selected timepoints. Green dots indicate transcripts that are up-regulated. Red dots indicate transcripts that are down-regulated. Grey dots indicate transcripts that are not significantly differentially expressed. A *q* value (adjusted *p *value) threshold of 0.01 was applied to determine statistical significance. The x-axis corresponds to log2FC between selected timepoints. The y-axis corresponds to − log10 of the *q *value (adjusted *p *value). Data-points corresponding to the most significantly differentially expressed transcripts (*q* value < 1E−40 and ranked by log2FC) with reaction associations in iDU1327 are circled. **A** Differential expression analysis between T1 and T2. The transcripts and their associated reactions that correspond to circled data-points are given in Table 1. **B** Differential expression analysis between T1 and T3. The transcripts and their associated reactions that correspond to circled data-points are given in Table 2. **C** Differential expression analysis between T2 and T3. The transcripts and their associated reactions that correspond to circled data-points are given in Table 3

To extend our investigation, we performed transcriptomic analysis at three key fermentation timepoints (Fig. 1). The first timepoint (T1) was taken, while external phosphate was still present before the onset of citric acid production and phosphate-limited growth. The other two timepoints (T2 and T3) were taken during citric acid production; the first of these (T2), while glucose was being consumed and the second (T3) during the main xylose consumption phase after glucose was fully consumed. Differential expression analysis revealed a greater degree of similarity between the two citric acid producing timepoints (T2 and T3) than for comparisons between these and the non-citric acid producing timepoint (T1) (Fig. 2).

**Fig. 3:**
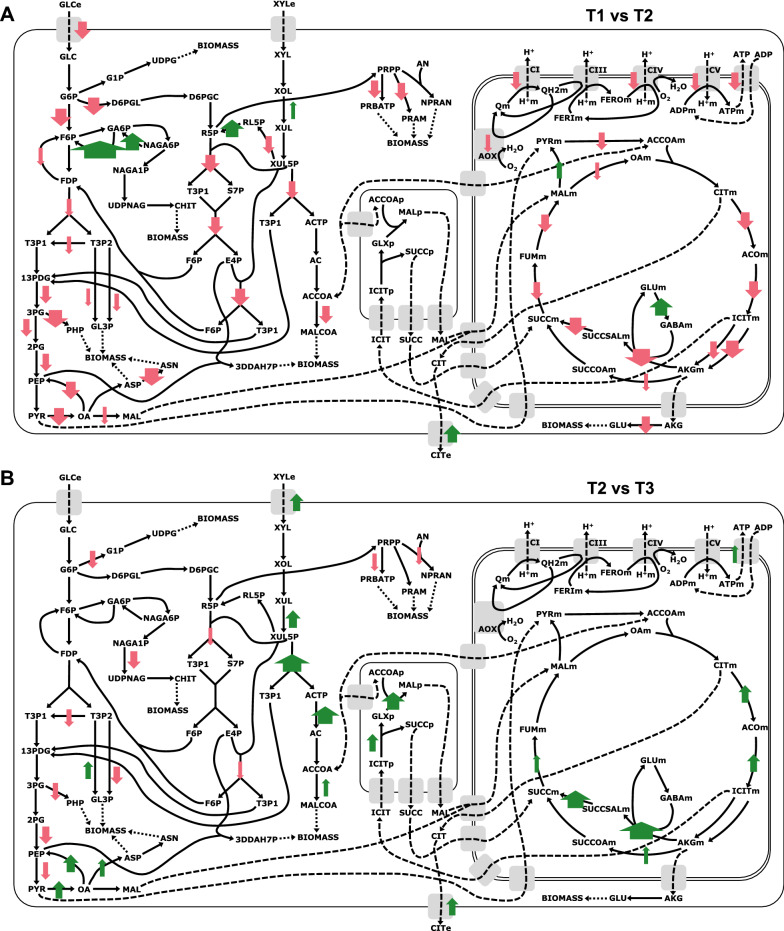
Metabolic schematics showing the key changes in the transcriptome from T1 to T2 (**A**) and from T2 to T3 (**B**) and their reaction-level effects. Green upward and red downward arrows indicate significantly up- and down-regulated reactions, respectively (*q* value < 0.01 and log2FC > 0.7). The arrow width is directly proportional to the log2FC value. The full names of abbreviated metabolites are given in the iDU1327 model (see Additional file 1). Reactions included are shown in simplified form with only key reactants and products

To enable us to identify potential in vivo constraints that limit citric acid production, we associated transcripts with the reactions in the metabolic model and determined expression at a reaction-level. The most differentially expressed transcripts with reaction associations are shown in Tables 1, 2, 3. With reaction-level expression determined, we constructed metabolic schematics to visualise the changes in the transcriptome and their reaction-level effects for a given comparison (Fig. 3).

**Table 1:** Most significantly differentially expressed transcripts between timepoints T1 and T2 with reaction associations in iDU1327

Transcript ID	Effect	Log2FC	Associated reactions in iDU1327	Names of associated reactions
Aspni_transcript.chr_202G213.1	Down-regulated	− 9.7	R462; R463; N1; R464	Catalase
Aspni_transcript.chr_202G683.1	Down-regulated	− 8.9	R623	5-Oxo-l-proline amidohydrolase (ATP-hydrolysing)
Aspni_transcript.chr_401G28.1	Down-regulated	− 8.1	R258; R259	Glucose oxidase
Aspni_transcript.chr_701G586.1	Down-regulated	− 6.4	R1227	Sulphite reductase
Aspni_transcript.chr_202G195.1	Down-regulated	− 6.1	R462; R463; N1; R464	Catalase
Aspni_transcript.chr_101G17.1	Down-regulated	− 5.5	R332; R333; R334	Alpha-galactosidase
Aspni_transcript.chr_304G54.1	Down-regulated	− 5	R74; R511	Formate oxidase; Phosphoglycerate dehydrogenase
Aspni_transcript.chr_302G484.1	Down-regulated	− 4.8	R305; R335; NR2	Fructan beta-fructosidase; Invertase; Stachyose fructohydrolase
Aspni_transcript.chr_301G287.1	Down-regulated	− 4.5	R477; R485; R1246	Argininosuccinate synthase; L-alanine:tRNA(Ala) ligase
Aspni_transcript.chr_101G512.1	Down-regulated	− 4.4	R417; R418	Chitinase
Aspni_transcript.chr_604G19.1	Up-regulated	10.8	R89; R90; R95; R96; R97; R98	Propanoate:CoA ligase (AMP-forming); Propionyl-CoA synthetase
Aspni_transcript.chr_402G104.3	Up-regulated	8.4	R377	Salicylate hydroxylase
Aspni_transcript.chr_202G947.1	Up-regulated	8.4	R35	Citrate synthase
Aspni_transcript.chr_601G472.1	Up-regulated	8.1	R398	4-Carboxymuconolactone decarboxylase
Aspni_transcript.chr_604G21.1	Up-regulated	8	R124; R131	Dihydrofolate synthase; Tetrahydrofolylpolyglutamate synthase
Aspni_transcript.chr_601G138.1	Up-regulated	7.4	R490	Acetylglutamate kinase
Aspni_transcript.chr_601G143.1	Up-regulated	7.2	R1187	Trans, trans-farnesyl-diphosphate:isopentenyl-diphosphate farnesyltranstransferase
Aspni_transcript.chr_402G104.6	Up-regulated	7.1	R377	Salicylate hydroxylase
Aspni_transcript.chr_503G231.2	Up-regulated	7	R411	Glucosamine-6-phosphate deaminase
Aspni_transcript.chr_603G120.1	Up-regulated	6.9	R791; R796; R801; R806; R811; R816; R821; R826; R831; R837; R842; R847; R852; R857; R862; R867; R872	3-Oxoacyl-[acyl-carrier-protein] reductase

Transcripts shown have *q* value < 1E−40 and are ranked by log2FC

**Table 2:** Most significantly differentially expressed transcripts between timepoints T1 and T3 with reaction associations in iDU1327

Transcript ID	Effect	Log2FC	Associated reactions in iDU1327	Names of associated reactions
Aspni_transcript.chr_601G340.1	Down-regulated	− 11.5	R544; R554	Dihydroxy acid dehydratase
Aspni_transcript.chr_202G213.1	Down-regulated	− 10.7	R462; R463; N1; R464	Catalase
Aspni_transcript.chr_202G683.1	Down-regulated	− 10.7	R623	5-Oxo-l-proline amidohydrolase (ATP-hydrolysing)
Aspni_transcript.chr_102G681.1	Down-regulated	− 10.6	R462; R463; N1; R464	Catalase
Aspni_transcript.chr_401G28.1	Down-regulated	− 9.3	R258; R259	Glucose oxidase
Aspni_transcript.chr_801G200.1	Down-regulated	− 8.8	R790; R795; R800; R805; R810; R815; R820; R825; R830; R836; R841; R846; R851; R856; R861; R866; R871	3-Oxoacyl-[acyl-carrier-protein] synthase
Aspni_transcript.chr_701G586.1	Down-regulated	− 7.4	R1227	Sulphite reductase
Aspni_transcript.chr_603G16.1	Down-regulated	− 6.9	R451	ATP synthase
Aspni_transcript.chr_302G588.1	Down-regulated	− 6.9	R719	Uracil phosphoribosyltransferase
Aspni_transcript.chr_202G195.1	Down-regulated	− 6.5	R462; R463; N1; R464	Catalase
Aspni_transcript.chr_402G104.3	Up-regulated	9.5	R377	Salicylate hydroxylase
Aspni_transcript.chr_601G472.1	Up-regulated	9.4	R398	4-Carboxymuconolactone decarboxylase
Aspni_transcript.chr_202G1357.1	Up-regulated	8.3	R362; R618; R619; R785; R1244; NR28; NR37	Benzonitrilase; Nitrilase; Formamide hydro-lyase; Phenylacetonitrile aminohydrolase
Aspni_transcript.chr_601G138.1	Up-regulated	8	R490	Acetylglutamate kinase
Aspni_transcript.chr_402G104.6	Up-regulated	7.7	R377	Salicylate hydroxylase
Aspni_transcript.chr_601G80.1	Up-regulated	7.6	R65; R66	Oxalate decarboxylase
Aspni_transcript.chr_601G143.1	Up-regulated	7.6	R1187	Trans,trans-farnesyl-diphosphate:isopentenyl-diphosphate farnesyltranstransferase
Aspni_transcript.chr_401G532.1	Up-regulated	7.5	R378; R402; R404; R615; NR23	Amine oxidase
Aspni_transcript.chr_503G231.2	Up-regulated	7.4	R411	Glucosamine-6-phosphate deaminase
Aspni_transcript.chr_304G666.1	Up-regulated	7.2	R211; R1115	Glycerol 3-phosphate dehydrogenase (NAD + dependent)

Transcripts shown have *q* value < 1E−40 and are ranked by log2FC

**Table 3:** Most significantly differentially expressed transcripts between timepoints T2 and T3 with reaction associations in iDU1327

Transcript ID	Effect	Log2FC	Associated reactions in iDU1327	Names of associated reactions
Aspni_transcript.chr_302G588.1	Down-regulated	− 8.9	R719	Uracil phosphoribosyltransferase
Aspni_transcript.chr_302G590.1	Down-regulated	− 7.3	R173	GTP 7,8–8,9-dihydrolase (diphosphate-forming)
Aspni_transcript.chr_401G344.1	Down-regulated	− 4.8	R1176	3-Hydroxy-3-methylglutaryl coenzyme A synthase
Aspni_transcript.chr_202G1142.1	Down-regulated	− 4.6	R193; R198	Alcohol dehydrogenase
Aspni_transcript.chr_802G171.1	Down-regulated	− 4.4	R228; R230; R791; R796; R801; R806; R811; R816; R821; R826; R831; R837; R842; R847; R852; R857; R862; R867; R872	L-Xylulose reductase;3-Oxoacyl-[acyl-carrier-protein] reductase
Aspni_transcript.chr_603G16.1	Down-regulated	− 4.1	R451	ATP synthase
Aspni_transcript.chr_102G146.1	Down-regulated	− 3.7	R791; R796; R801; R806; R811; R816; R821; R826; R831; R837; R842; R847; R852; R857; R862; R867; R872	3-Oxoacyl-[acyl-carrier-protein] reductase
Aspni_transcript.chr_101G224.1	Down-regulated	− 3.3	R322	alpha-amylase
Aspni_transcript.chr_304G378.1	Down-regulated	− 3.2	R791; R796; R801; R806; R811; R816; R821; R826; R831; R837; R842; R847; R852; R857; R862; R867; R872	3-Oxoacyl-[acyl-carrier-protein] reductase
Aspni_transcript.chr_801G344.1	Down-regulated	− 3	R182; R188; R265; R1207; NR14; NR26	Riboflavin-5-phosphate phosphohydrolase; Thiamin monophosphate phosphohydrolase; Phosphatidate phosphatase; 4-Nitrophenyl phosphate phosphohydrolase; Glycerone phosphate phosphohydrolase
Aspni_transcript.chr_304G666.1	Up-regulated	4.8	R211; R1115	Glycerol 3-phosphate dehydrogenase (NAD + dependent)
Aspni_transcript.chr_101G504.1	Up-regulated	4.7	R71; R75; R516	S-(hydroxymethyl)glutathione dehydrogenase; Formaldehyde dehydrogenase; Threonine dehydrogenase
Aspni_transcript.chr_602G271.2	Up-regulated	4.7	R107; R108	Succinate-semialdehyde dehydrogenase
Aspni_transcript.chr_501G182.1	Up-regulated	4.6	R106; R611	4-Aminobutyrate transaminase
Aspni_transcript.chr_102G293.1	Up-regulated	4.5	R107; R108	Succinate-semialdehyde dehydrogenase
Aspni_transcript.chr_202G964.1	Up-regulated	3.8	R153; R479; R604	Adenosyl:methionine-8-amino-7-oxononanoate aminotransferase; Ornithine transaminase
Aspni_transcript.chr_101G108.1	Up-regulated	3.7	R378; R402; R404; R615; NR23	Amine oxidase
Aspni_transcript.chr_402G585.2	Up-regulated	3.7	R33; R34	Phosphoketolase
Aspni_transcript.chr_202G803.1	Up-regulated	3.6	R103; R104	Methylmalonate-semialdehyde dehydrogenase
Aspni_transcript.chr_402G613.1	Up-regulated	3.1	R78	Pyruvate decarboxylase

Transcripts shown have *q* value < 1E−40 and are ranked by log2FC

In comparing T1 with T2, the scale of change is clear when transitioning to citric acid production with widespread differential expression events observed across metabolism (Fig. 3A). In particular, reactions involved in biomass production were down-regulated, while citrate export was up-regulated together with the down-regulation of TCA cycle reactions involved in citrate catabolism. Unexpectedly, pyruvate carboxylase whose activity is important to citric acid production [18] was down-regulated, suggesting this step as a point of constraint in vivo.

The expression changes are less extensive when transitioning from glucose to xylose consumption and appear to be directed at the change in substrate use (Fig. 3B). These include up-regulation of xylose import and xylulose kinase as well as phosphoketolase and acetate kinase that appear to activate an alternative xylose catabolic pathway, which may be associated with up-regulation of the glyoxylate shunt through an increased supply of acetyl-CoA. We also observed further up-regulation of citrate export, yet the rate of citric acid production in silico is around 2.3 times higher at T2 than T3 when citric output is unconstrained (Table 4). The lower citrate exporter expression at T2 with respect to T3 may indicate citrate export as a point of constraint in vivo.

**Table 4:** Input/output fluxes in iDU1327 at selected timepoints without constraint on citric output

Input/output reaction	T1 flux (mmol gDW^−1^ h^−1^)	T2 flux (mmol gDW^−1^ h^−1^)	T3 flux (mmol gDW^−1^ h^−1^)
Glucose (DGLCe <==>)	− 1.2552	− 0.4392	0.0
Xylose (XYLe <==>)	− 0.0718	− 0.0647	− 0.1936
External phosphate (PIe <==>)	− 0.1455	0.0	0.0
Internal phosphate (PI <==>)	0.1339	− 0.0019	− 0.00005
Biomass	0.1207 (h^−1^)	0.0195 (h^−1^)	0.0006 (h^−1^)
Citric acid (CIT-e <==>)	0.0	0.3159	0.135
Carbon dioxide (CO_2_e <==>)	3.3671	0.3311	0.137
Oxygen (O_2_e <==>)	− 1.9835	− 0.581	− 0.3331

### Investigating suboptimal citric acid production by transcriptome-guided in silico evolution

**Fig. 4:**
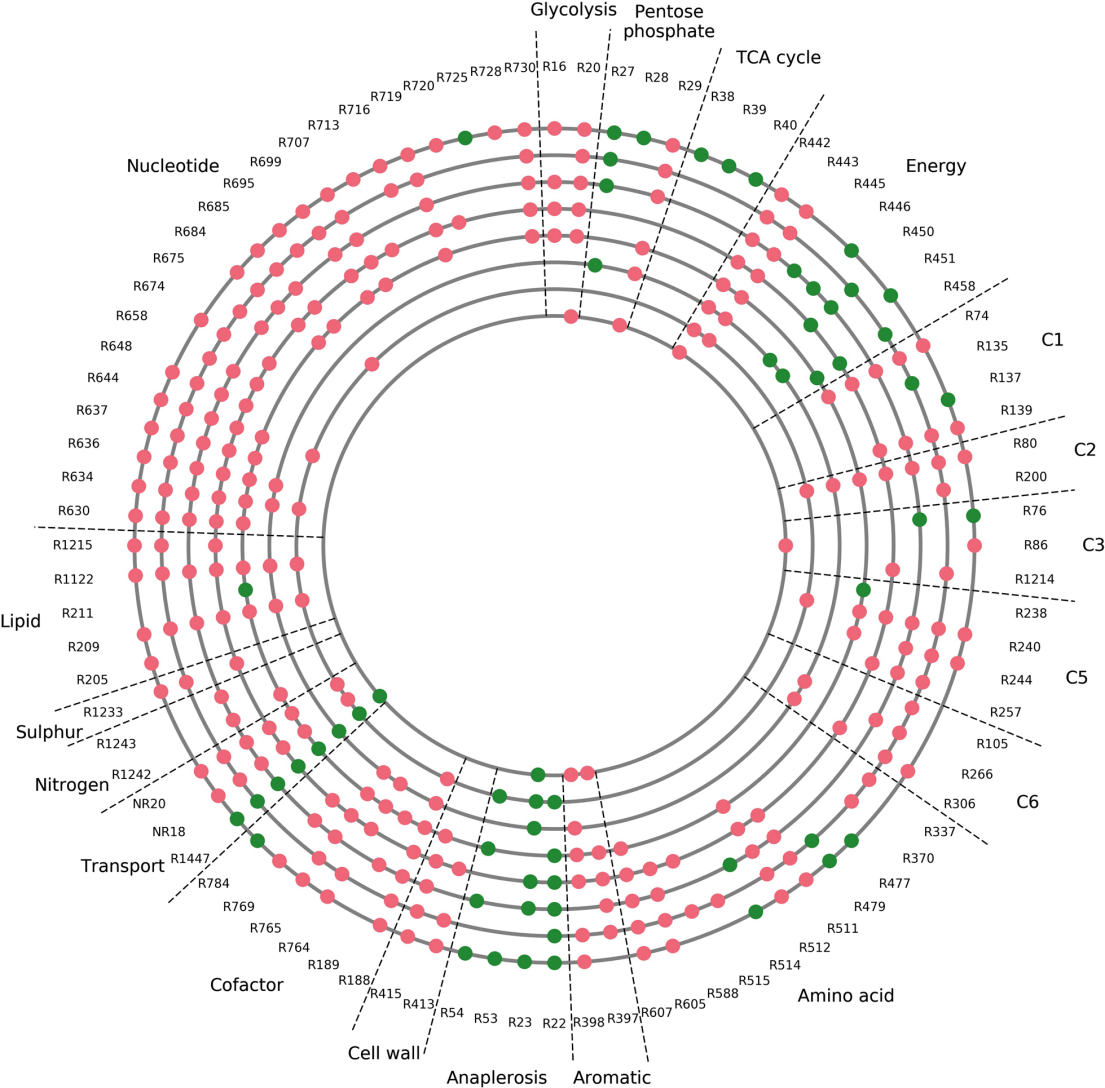
Plot giving a comparative view of the target suggestions from 8 independent runs of the evolutionary algorithm. Each of the eight grey circles corresponds to the results of one replicate run, and dots on these circles indicate which reactions were targeted in the given run. The IDs of the reactions targeted are shown on the outside (see Table 6 for corresponding reactions). Green and red dots indicate targets for up- and down-regulation, respectively. The sectors indicate the areas of metabolism that were targeted

To develop a metabolic understanding of suboptimal citric acid production, we developed an evolutionary algorithm to perform in silico evolution of the model with the aim of reflecting non-optimised strains. We focused on T2 as citric output is around 2.6 times higher at T2 when unconstrained (Table 4) than when constrained (Table 5) to fit in vivo data, whereas citric output at T3 is virtually the same. The objective was to identify changes to flux bounds that constrain citric output to the value that closely fits in vivo data while maintaining the same carbon input and biomass output. To achieve this, we adapted an evolutionary algorithm [19] to evolve the model to more accurately reflect the in vivo metabolic state that is associated with constrained citric production. As many solutions may exist to this, we used the transcriptomic data to guide the in silico evolution to limit solutions to those that are more likely to resemble the one indicated by the transcriptome. This constrained the evolutionary algorithm to alter flux bounds only on reactions where there is a significant differential expression, with such cases implying transcriptional regulation over the reaction’s activity. We compared and analysed the solutions from eight independent runs of the evolutionary algorithm to suggest targets for increasing citric acid productivity (Fig. 4). In total, we found 91 reactions suggested for targeted intervention of their activity; 65 for down-regulation and 26 for up-regulation (Table 6). Together, the list of targets provides high coverage of all potential targets that could bring about optimal citric acid production. Some of the targets were expected and consistent with the literature for example the up-regulation of citrate export and pyruvate carboxylase, while other targets were novel such as the down-regulation of inorganic diphosphatase.

**Table 5:** Input/output fluxes in iDU1327 at selected timepoints with citric output constrained in line with in vivo data

Input/output reaction	T1 flux (mmol gDW^−1^ h^−1^)	T2 flux (mmol gDW^−1^ h^−1^)	T3 flux (mmol gDW^−1^ h^−1^)
Glucose (DGLCe <==>)	− 1.2552	− 0.4392	0.0
Xylose (XYLe <==>)	− 0.0718	− 0.0647	− 0.1936
External phosphate (PIe <==>)	− 0.1455	0.0	0.0
Internal phosphate (PI <==>)	0.1339	− 0.0019	− 0.00005
Biomass	0.1207 (h^−1^)	0.0195 (h^−1^)	0.0006 (h^−1^)
Citric acid (CIT-e <==>)	0.0	0.12	0.12
Carbon dioxide (CO_2_e <==>)	3.3671	1.5066	0.2268
Oxygen (O_2_e <==>)	− 1.9835	− 1.4626	− 0.4004

**Table 6:** Suggested targets for increasing citric acid output based on results from the evolutionary algorithm (primarily ranked by frequency and then by citric %increase)

Reaction ID^a^	Name	Equation	Target	Frequency^b^	Citric %increase^c^
R1447	Citrate exporter	CIT-e ↔ CIT	Up-regulate	8	163
R442	Diphosphatase	PPI + H_2_O → 2*PI + H	Down-regulate	8	163
R209	Glycerol 3-phosphate dehydrogenase (FAD dependent)	GL3P + FADm → T3P2 + FADH2m	Down-regulate	7	170
R634	Phosphoribosyl amino imidazolesuccinocarbozamide synthetase	ASP + ATP + CAIR ↔ 2*H + PI + ADP + SAICAR	Down-regulate	7	163
R80	Acetate kinase	ATP + AC ↔ ADP + ACTP	Down-regulate	7	163
R188	Thiamin monophosphate phosphohydrolase	THMP + H_2_O → THM + PI	Down-regulate	7	163
R443	Diphosphatase	PPIm + H_2_O → 2*PIm + Hm	Down-regulate	7	163
R1122	ATP:ethanolamine O-phosphotransferase	ATP + ETHLA → ADP + PEA + H	Down-regulate	7	163
R22	Pyruvate carboxylase	ATP + PYR + H_2_O + CO_2_ → ADP + PI + OA + 2*H	Up-regulate	6	171
R636	IMP cyclohydrolase	AICAR + FTHF ↔ THF + PRFICA	Down-regulate	6	169
R398	4-Carboxymuconolactone decarboxylase	4CMUCL + 2*H → OAEL + CO_2_	Down-regulate	6	163
R20	Phosphopyruvate hydratase	2PG ↔ PEP + H_2_O	Down-regulate	6	163
NR18	Acetyl-CoA:carnitine O-acetyltransferase	ACCOAm + CARm ↔ COAm + ALCARm	Down-regulate	6	163
R23	Pyruvate carboxylase	ATPm + PYRm + H_2_Om + CO_2_m → ADPm + PIm + OAm + 2*Hm	Up-regulate	6	163
NR20	Acetyl-CoA:carnitine O-acetyltransferase	ACCOA + CAR ↔ COA + ALCAR	Down-regulate	6	163
R29	Ribose-5-phosphate isomerase	R5P ↔ RL5P	Down-regulate	6	163
R765	Nicotinate phosphoribosyltransferase	NICA + PRPP → NAMN + PPI	Down-regulate	6	162
R240	Phosphoglucomutase	R5P ↔ R1P	Down-regulate	6	162
R685	Allantoicase	ATT + H_2_O ↔ UGC + UREA	Down-regulate	6	161
R644	Nucleoside-diphosphate kinase	ATP + DADP ↔ ADP + DATP	Down-regulate	6	161
R764	Nicotinamidase	NICD + H_2_O ↔ NICA + NH3	Down-regulate	5	164
R397	3-Carboxy-cis,cis-muconate cycloisomerase	3CMUCO ↔ 4CMUCL	Down-regulate	5	163
R415	UDP-N-acetylglucosamine pyrophosphorylase	UTP + NAGA1P ↔ PPI + UDPNAG	Down-regulate	5	163
R512	Phosphoserine transaminase	PHP + GLU → AKG + 3PSER	Down-regulate	5	163
R607	Proline dehydrogenase	NADm + PROm → 2*Hm + NADHm + P5Cm	Down-regulate	5	163
R684	Allantoinase	ATN + H_2_O ↔ ATT	Down-regulate	5	163
R707	Nucleoside-diphosphate kinase	DUDP + ATP ↔ DUTP + ADP	Down-regulate	5	163
R74	Formate oxidase	FOR + O_2_ + H → H_2_O_2_ + CO_2_	Down-regulate	5	163
R637	IMP cyclohydrolase	PRFICA ↔ H_2_O + IMP	Down-regulate	5	163
R699	Nucleoside-diphosphate kinase	CTP + ADP ↔ CDP + ATP	Down-regulate	5	162
R630	Phosphoribosylglycinamide formyltransferase	FTHF + GAR → H + THF + FGAR	Down-regulate	5	161
R719	Uracil phosphoribosyltransferase	URA + PRPP → UMP + PPI + H	Down-regulate	5	160
R730	Cytosine deaminase	CYTS + H_2_O + H → URA + NH_3_	Down-regulate	5	147
R306	Ketohexokinase	ATP + FRU → ADP + F1P + H	Down-regulate	4	163
R648	Nucleoside-diphosphate kinase	ATP + GDP ↔ ADP + GTP	Down-regulate	4	163
R605	Pyrroline-5-carboxylate reductase	2*H + NADPH + P5C ↔ PRO + NADP	Down-regulate	4	163
R244	Ribulokinase	ATP + RL → ADP + RL5P + H	Down-regulate	4	163
R139	5-Formyltetrahydrofolate deformylase	FTHF + H_2_O → FOR + THF + H	Down-regulate	4	162
R511	Phosphoglycerate dehydrogenase	NAD + 3PG ↔ H + NADH + PHP	Down-regulate	4	162
R16	Triosephosphate isomerase	T3P2 ↔ T3P1	Down-regulate	4	144
R27	Phosphogluconate dehydrogenase	D6PGC + NADP → RL5P + CO_2_ + NADPH	Up-regulate	4	111
R54	Malate synthase	ACCOAp + H2Op + GLXp → MALp + COAp + Hp	Up-regulate	4	104
R450	Cytochrome c oxidase	2*FEROm + 0.5*O_2_m + 6*Hm → 2*FERIm + H_2_Om + 4*Ho	Up-regulate	4	91
R658	Purine nucleoside hydrolase	ADN + H_2_O → AD + RIB	Down-regulate	3	163
R257	D-arabinitol 2-dehydrogenase(NAD +)	AOL + NAD → RL + NADH + H	Down-regulate	3	163
R1215	D-Glyceraldehyde:NAD + oxidoreductase	G + NADH + 2*H ↔ GLYAL + NAD + H_2_O	Down-regulate	3	163
R1233	3'—5' Bisphosphate nucleotidase	PAP + H_2_O → AMP + PI	Down-regulate	3	162
R674	Purine nucleosidase	GSN + H_2_O → GN + RIB	Down-regulate	3	162
R769	NAD synthetase	ATP + DMNAD + GLN + H_2_O ↔ AMP + PPI + NAD + GLU + 2*H	Down-regulate	3	162
R266	Gluconokinase	GLCNT + ATP → D6PGC + ADP + H	Down-regulate	3	131
R413	N-acetylglucosamine-6-phosphate deacetylase	NAGA6P + H_2_O → GA6P + AC	Down-regulate	3	126
R458	ADP/ATP translocase	ADP + PI + ATPm + H2Om → ADPm + PIm + ATP + H_2_O	Up-regulate	3	111
R720	dUTP pyrophosphatase	DUTP + H_2_O → PPI + DUMP + 2*H	Down-regulate	2	163
R675	Guanine aminohydrolase	GN + H_2_O ↔ XAN + NH_3_	Down-regulate	2	163
R1242	Urea carboxylase	UREA + ATP + H_2_O + CO_2_ ↔ ADP + PI + UREAC + 2*H	Down-regulate	2	163
R189	ATP:thiamine diphosphotransferase	ATP + THM → AMP + THDP + 2*H	Down-regulate	2	163
R1214	Glycerate 3-kinase	ATP + G → ADP + 3PG + H	Down-regulate	2	162
R86	Lactoylglutathione lyase	RGT + MTHGXL ↔ LGT	Down-regulate	2	144
R716	Uridine kinase	URI + GTP → UMP + GDP + H	Down-regulate	2	134
R588	Chorismate mutase	CHOR → PHEN	Down-regulate	2	130
R695	Nucleoside-diphosphate kinase	UDP + ATP ↔ UTP + ADP	Down-regulate	2	127
R446	Respiratory-chain NADH dehydrogenase	NADH + Qm + 5*Hm → NAD + QH2m + 4*Ho	Up-regulate	2	107
R479	Ornithine transaminase	ORN + AKG → GLUGSAL + GLU	Up-regulate	2	93
R514	Glycine hydroxymethyltransferase	THF + SER ↔ H_2_O + GLY + METTHF	Up-regulate	2	90
R713	ADP-ribose pyrophosphatase	ADPR + H_2_O → AMP + R5P + 2*H	Down-regulate	2	90
R451	ATP synthase	ADPm + PIm + 4.5454*Ho → ATPm + H_2_Om + 4.5454*Hm	Up-regulate	2	67
R76	Pyruvate dehydrogenase	PYRm + NADm + COAm → ACCOAm + NADHm + CO_2_m + Hm	Up-regulate	2	65
R105	Glutamate decarboxylase	GLUm + Hm → GABAm + CO_2_m	Down-regulate	1	173
R515	Threonine aldolase	GLY + ACAL ↔ THR	Down-regulate	1	163
R370	Kynurenine formamidase	FKYN + H_2_O → FOR + KYN + H	Down-regulate	1	154
R1243	Allophanate hydrolase	UREAC + H_2_O → 2*NH_3_ + 2*CO_2_	Down-regulate	1	153
R200	Aldehyde dehydrogenase (NADP+)	ACALm + NADPm + H_2_Om → ACm + NADPHm + 2*Hm	Down-regulate	1	133
R135	Methylenetetrahydrofolate dehydrogenase (NADP +)	METHFm + NADPHm ↔ METTHFm + NADPm	Up-regulate	1	116
R784	5'-Nucleotidase	NAMN + H_2_O → NAR + PI	Up-regulate	1	115
R728	Cytidine deaminase	CYTD + H_2_O → URI + NH_3_	Down-regulate	1	114
R211	Glycerol 3-phosphate dehydrogenase (NAD+ dependent)	H + T3P2 + NADH → GL3P + NAD	Up-regulate	1	113
R337	Phenylacetaldehyde dehydrogenase	PHAL + NADP + H_2_O ↔ PHAC + NADPH + 2*H	Down-regulate	1	113
R205	Glycerol dehydrogenase	H + GLYAL + NADPH → GL + NADP	Down-regulate	1	112
R53	Isocitrate lyase	ICITp → SUCCp + GLXp	Up-regulate	1	111
R445	Respiratory-chain NADH dehydrogenase	NADHm + Qm + 5*Hm → NADm + QH2m + 4*Ho	Up-regulate	1	103
R28	Ribulose-phosphate 3-epimerase	RL5P ↔ XUL5P	Up-regulate	1	100
R40	Isocitrate dehydrogenase (NADP+)	ICIT + NADP → AKG + CO2 + NADPH	Up-regulate	1	96
R39	Isocitrate dehydrogenase (NAD+)	ICITm + NADm → AKGm + CO2m + NADHm	Up-regulate	1	96
R725	5'-Nucleotidase	UMP + H_2_O → PI + URI	Up-regulate	1	95
R137	Methylenetetrahydrofolate dehydrogenase (NAD+)	METTHF + NAD → METHF + NADH	Up-regulate	1	92
R38	Isocitrate dehydrogenase (NADP+)	ICITm + NADPm → AKGm + CO_2_m + NADPHm	Up-regulate	1	63
R477	Argininosuccinate synthase	ASP + ATP + CITR → 2*H + AMP + PPI + ARGSUCC	Up-regulate	1	53
R238	Dihydroxyacetone synthase	XUL5P + FALD ↔ T3P1 + GLYN	Up-regulate	1	53
R376	4-Hydroxyphenylpyruvate dioxygenase	4HPP + O_2_ → HOMOGEN + CO_2_	Down-regulate	N/A	150
R78	Pyruvate decarboxylase	H + PYR → ACAL + CO_2_	Up-regulate	N/A	80
R199	Aldehyde dehydrogenase (NAD+)	ACALm + NADm + H2Om → ACm + NADHm + 2*Hm	Down-regulate	N/A	57

^a^The ID of the reaction in the iDU1327 metabolic model (see Additional file 1)

^b^The number of runs of in silico evolution the target occurred in. Where the frequency is marked as N/A, the target occurred in a subsequent run with mutations disallowed on previously targeted reactions

^c^The percentage increase in citric acid output flux at T2 when the flux bounds of the reaction are set to their original unconstrained values while retaining the constrained flux bounds of the other reactions in the solution. If the given reaction is present in multiple solutions, the highest percentage increase is given

## Discussion

In our in vivo fermentation experiments with sugarcane bagasse hydrolysate, we observed a promising yield of citric acid; up to 50 g/L in 6 days from 80 g/L glucose and 40 g/L xylose. In our simulations, however, up to 85 g/L citric acid could be produced. By our analysis of the transcriptome at key timepoints and with our in silico toolkit, we have determined what may underlie the suboptimal citric acid production. The exhaustive list of targets all involve a common feature: an aim to minimise carbon loss as CO_2_ and maximise citric output.

One example of a target that is associated with minimising carbon loss via CO_2_ is the down-regulation of inorganic diphosphatase. Forcing flux of this reaction alone was able to decrease citric output to the target value, suggesting that a high level of inorganic diphosphatase activity may negatively affect citric acid production. The reaction catalysed by inorganic diphosphatase acts to dissipate energy, thereby supporting a high carbon input flux with carbon output predominantly to CO_2_. This finding also relates to our previous work [17] on the relationship between phosphate levels and citric acid production. Decreased activity of inorganic diphosphatase may limit internal phosphate levels and enhance citric acid production. The majority of our targets for down-regulation are associated with anabolic pathways involved in the synthesis of biomass components. As the production of biomass becomes restricted by phosphate availability during citric acid production, any excess in anabolic flux would result in futile pathways. Comparison of the biomass output flux values between T2 and T3 reveals that the growth rate is ≈30-fold higher at T2, yet the fold changes in expression of anabolic reactions are significantly less than the fold change in growth rate, suggesting that the expression of these reactions is not excessive at T2.

Among our targets are expected changes in metabolism including the up-regulation of citrate export and pyruvate carboxylase, both of which have significantly lower expression at T2 with respect to T3. The flux through these steps would be higher at T2 than T3 in the case of optimal citric acid production, suggesting that expression should also be higher at T2 contrary to what we see in this study. The citrate exporter has been overexpressed previously which resulted in a fivefold increase in citric acid production [20], and pyruvate carboxylase has been overexpressed for increasing production of malic acid [21].

The importance of energy metabolism to citric acid production is highlighted by the frequent targeting of oxidative phosphorylation reactions. These reactions were down-regulated from T1 to T2 by around 2–2.6-fold, and constraining the flux of these reactions in line with the transcriptome data led to a drop in citric production. This may seem counter-intuitive as the addition of oxidative phosphorylation inhibitors has been shown to increase citric acid production; however, negative effects were observed when the activity of oxidative phosphorylation was too low [22]. This is consistent with our study, which shows that over-constraint of oxidative phosphorylation decreases citric output.

The objective of our study was to identify targets for increasing citric acid production by integrating transcriptome data with metabolic modelling. Many efforts have been made to integrate transcriptome data with metabolic models, with early examples including the GIMME algorithm [10], E-Flux [11], and iMAT [13], and more recently SPOT [15]. A disadvantage of these approaches was their use of absolute expression data that may not correlate closely with reaction activity. An alternative is to use differential expression data that indicate which reactions are subject to transcriptional regulation, such as MADE where differential expression data are used to determine binary expression states [14]. Other methods include PROM that requires a regulatory network [12] and LBFBA that relies on flux data to parameterise linear reaction-specific functions to determine flux bounds from expression data [16]. Our approach infers from differential expression data the metabolic factors that underpin suboptimal citric acid production in *Aspergillus*, and is tailored to applications where there is a defined metabolic goal. Its basis is an evolutionary algorithm with changes to flux bounds guided by differential expression data. Its limitation is that it outputs a set of possible solutions rather than a unique solution.

## Conclusions

In this study, we demonstrate the production of citric acid from lignocellulosic hydrolysate by an engineered variant of *A. niger* ATCC1015. By performing in silico simulations using a dynamic model, we show how transcriptomic data across multiple timepoints can be coupled with evolutionary and metabolic modelling to inform targeted engineering strategies aimed at maximising productivity from a chosen feedstock. The same in silico strategies employed here can be applied to other biotechnological goals, assisting efforts to harness the potential of microorganisms for bio-based production of valuable chemicals.

## Methods

### Preparation of sugarcane bagasse hydrolysate

Sugarcane bagasse was obtained from Natems Sugar Pvt. Ltd. (India) and dried at 50 °C overnight to reach constant weight. Bagasse was milled using a knife mill with a 1 mm sieve prior to pre-treatment. Pre-treatment was performed in a 2 L vessel (Parr Instrument Company, Moline, IL, US): 100 g milled bagasse was added to the vessel and mixed with 900 mL 0.4 M NaOH to homogeneity. The vessel was heated to 140 °C and maintained at 140 °C for 45 min, and then cooled on ice until the temperature dropped to 60 °C. The contents of the vessel were transferred to a fruit press after pre-treatment. Pre-treated bagasse was pressed to remove the pre-treatment liquor and rinsed twice in 500 mL acidified dH_2_O. The acidified dH_2_O was prepared by adding 100 µl concentrated H_2_SO_4_ to 1.2 L dH_2_O. After rinsing, pre-treated bagasse was adjusted to pH 5–6 by the addition of concentrated H_2_SO_4_. The pre-treated bagasse was then transferred to Weck jars and autoclaved (121 °C 15 min) followed by storage at 4 °C until use. The pre-treated bagasse was subjected to enzymatic hydrolysis in 1 L shake flasks: Pre-treated bagasse was added to the flask at the equivalent of 50 g dry weight and autoclaved. Under aseptic conditions, 10 mL 1 M MES buffer pH 5.5 (filter sterile) and 24.5 mL enzyme solution (filter sterile) were added, followed by sterile dH_2_O up to a final volume of 400 mL. Enzyme solution was prepared by mixing 20 g Cellic CTec3 (Novozymes) with 20 g 25 mM MES buffer pH 5.5. Flasks were incubated at 50 °C with shaking at 160 rpm for 48 h. After hydrolysis, the hydrolysate slurry was centrifuged at 4600 rpm for 20 min in a Multifuge 3 SR benchtop centrifuge (Heraeus, Germany). The clear supernatant was filtered through Whatman glass microfibre filters GF/F (GE Healthcare UK Ltd., UK) using a vacuum pump and then filter sterilised into a sterile glass bottle using a Stericap™ PLUS filter (Merck Millipore). The filter sterile hydrolysate was stored at 4 °C.

### Shake flask fermentation experiments with time-course sampling

Fermentation experiments were performed in 250 mL baffled shake flasks (Bellco Glass Inc.; Vineland, NJ, USA) at a working volume of 30 mL. Bagasse hydrolysate was supplemented with 3 g/L NaNO_3_ and 10 mM uridine. Spores from the *A. niger* strain ATCC1015 Δ*pyrG* Δ*oah* Δ*gox* [17] were added at 1 × 10^6^ spores/mL. Spores were harvested from potato dextrose agar slants supplemented with 10 mM uridine. The slants were incubated at 37 °C for 3 days and spores were harvested using sterile cotton wool buds. Spores were suspended in saline Tween (0.1% Tween 80, 9 g/L NaCl) and centrifuged at 2500 rpm for 5 min. Spores were then washed 3 times in saline Tween prior to being used to inoculate cultures. Cultures were incubated at 30 °C with shaking at 250 rpm for 8 days. 500 µl homogeneous samples were taken twice daily 6 h apart. The supernatant and the biomass were separated by centrifugation at 20238 g for 3 min and stored at − 20 °C to prevent any changes to metabolite concentrations in the supernatant and any changes to biomass dry weight.

### Extracellular metabolite and biomass dry weight analysis

Glucose, xylose, and citric acid were determined enzymatically using the K-GLUC, K-XYLOSE, and K-CITR kits, respectively (Megazyme International Ireland Ltd., Wicklow, Ireland). Phosphate was determined using an assay kit (ab65622; Abcam, Cambridge, UK). Biomass dry weight was determined by washing biomass samples in pre-dried, pre-weighed 1.5 mL Eppendorf tubes 7 times in 1 mL dH_2_O, followed by drying at 70 °C to constant weight. Between each of the washing steps, the biomass samples were centrifuged at 20238 g for 3 min to pellet the biomass, and the supernatant was aspirated without disruption of the biomass pellet.

### Dynamic modelling to simulate the fermentation of bagasse hydrolysate to citric acid

Dynamic modelling was done as described previously [17] with some modifications. In brief, the FBA calculations were performed using bespoke Java code which implements the GLPK toolkit (GNU). dFBA routines were written directly into the Java code with the differential equations representing transport reactions solved by simple time-stepping (Euler method) with small values for the time-step. The iDU1756 model [19] was used, and deletions of the *pyrG*, *gox*, and *oah* genes were simulated by setting the flux bounds of their corresponding reactions to zero. Nitrate was used as the nitrogen input and uridine input was enabled. The sequential uptake of glucose and xylose was modelled by disabling xylose transport-mediated uptake at external glucose concentrations above 5 mM; the threshold of 5 mM was applied as this gave the best fit to the in vivo data. The kinetic parameters applied in the model are given in Table 7. The dFBA start time was adjusted to 10 h after inoculation.

**Table 7:** Kinetic parameters applied in dynamic modelling of sugarcane bagasse hydrolysate fermentation

Parameter	Description	Value
$v_{{Pe,{\text{max}}}}$ (mmol gDW^−1^ h^−1^)	External phosphate maximum input rate	0.15
$K_{Pe}$ (mM)	External phosphate Michaelis constant	0.0333^a^
$v_{{P,{\text{max}}}}$ (mmol gDW^−1^ h^−1^)	Internal phosphate maximum input rate	0.06
$K_{P}$ (mM)	Internal phosphate Michaelis constant	20
$v_{G1}$ (mmol gDW^−1^ h^−1^)	External glucose passive uptake rate	0.0027 × [GLC]^b^
$v_{{G2,{\text{max}}}}$ (mmol gDW^−1^ h^−1^)	External glucose transport-mediated uptake maximum rate	0.08
$K_{G2}$ (mM)	External glucose transport-mediated uptake Michaelis constant	0.26^a^
$K_{i2}$ (mM)	External glucose transport-mediated uptake citrate inhibition constant	933^a^
$v_{X1 }$ (mmol gDW^−1^ h^−1^)	External xylose passive uptake rate	0.00027 × [XYL]^c^
$v_{{X2,{\text{max}}}}$ (mmol gDW^−1^ h^−1^)	External xylose transport-mediated uptake maximum rate	0.18
$K_{X2}$ (mM)	External xylose transport-mediated uptake Michaelis constant	3.33^a^
$v_{{{\text{CIT}}}}$ (mmol gDW^−1^ h^−1^)	Citric acid output rate constraint^d^	0.12^a^

^a^These values are the same as in the dynamic modelling reported previously [17]

^b^[GLC] is the concentration of external glucose in mM

^c^[XYL] is the concentration of external xylose in mM

^d^Citric acid output rate constraint was only applied 32 h after the start time

### Sampling for transcriptome analysis and isolation of RNA for RNA-Seq

Cultures were setup as described previously. For the timepoints T1, T2, and T3, cultures were harvested in biological triplicates at 21, 72, and 132 h, respectively. Cultures were harvested as follows: The flask contents were filtered through a double layer of Miracloth to separate the mycelia from the culture liquid. The mycelia were washed 2 times in chilled 100 mM Tris.HCl buffer pH 7.5 (≈150 mL per wash) and then 3 times in chilled dH_2_O (≈150 mL per wash). Washed mycelia were squeeze-dried in Miracloth and transferred to 50 mL Falcon tubes on ice and then flash frozen in liquid nitrogen followed by storage at − 80 °C. Samples were freeze-dried overnight prior to use for RNA isolation and stored at − 80 °C thereafter. RNA was extracted and purified: For each sample, 5 mg freeze-dried mycelia were added to a pre-cooled 2 mL Eppendorf tube with two 3 mm tungsten carbide beads. The tubes were dipped in liquid nitrogen and kept on ice. Freeze-dried mycelia were ground using a TissueLyser set to 30 Hz for 30 s four times. 1 mL TRIzol reagent was added to each sample of ground mycelia followed by agitation using a TissueLyser set to 30 Hz for 30 s four times. RNA was extracted by the TRIzol method (Thermo Fisher Scientific) according to the manufacturer’s instructions. RNA pellets were air-dried at 37 °C for 10 min and solubilised in 200 µl RNase-free water at 60 °C for 15 min. RNA samples were stored at − 80 °C. RNA samples were further purified using the TURBO DNA-free™ kit (Thermo Fisher Scientific) according to the manufacturer’s instructions. RNA samples were sent to the University of York Technology Facility for RNA-Seq library preparation by poly(A) purification, and libraries were sequenced at the University of Leeds Next Generation Sequencing Facility using an Illumina HiSeq 3000 platform with 2 × 150 bp sequencing.

### Bioinformatics processing of RNA-Seq data

**Fig. 5:**
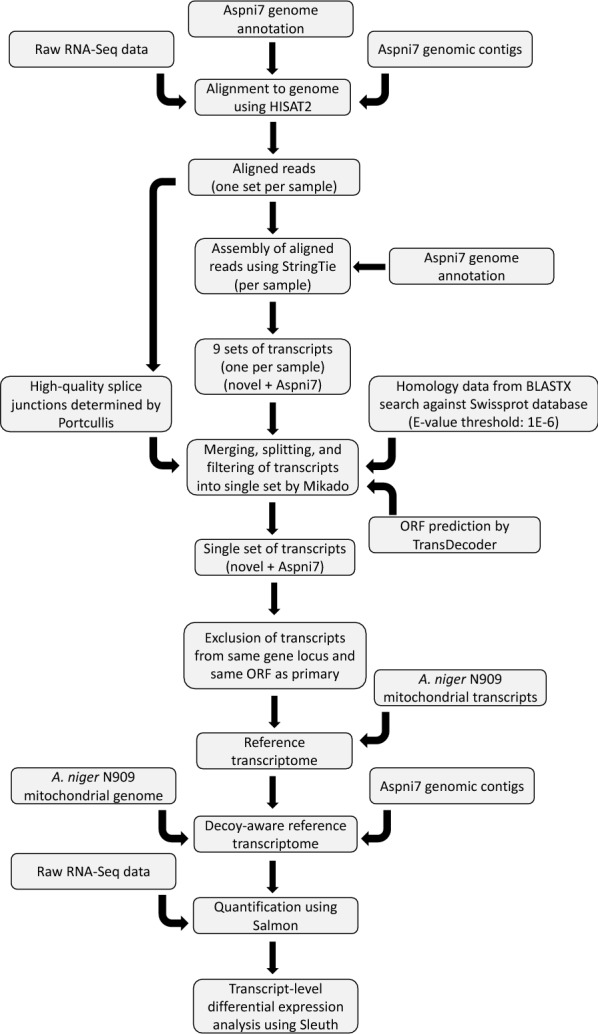
Schematic showing RNA-Seq analysis workflow

The tools used to construct the reference transcriptome were HISAT2 [23], StringTie [24], Mikado [25], Portcullis [26], and TransDecoder [27]. The latest version (v 4.0) of the *A. niger* ATCC1015 genome annotation [28] available from the Joint Genome Institute was used. The mitochondrial transcripts determined for the *A. niger* strain N909 [29] were included in the reference transcriptome. The tools used to perform quantification and differential expression analysis were Salmon [30], Wasabi [31], and Sleuth [32]. Figure 5 shows the workflow followed to process the RNA-Seq data.

### Functional annotation of transcripts and generation of transcript–reaction associations for the iDU1327 model

**Fig. 6:**
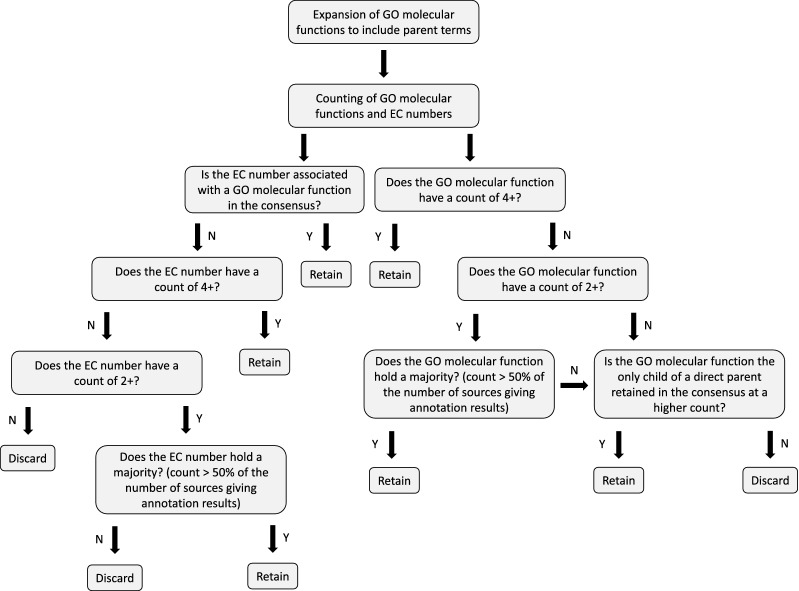
Schematic showing workflow followed to construct consensus functional annotation

The gene–protein–reaction associations in the iDU1756 model [19] were replaced with transcript–reaction associations in the model iDU1327 (see Additional file 1), determined by a comprehensive functional annotation process that employed a multitude of tools (Table 8). Mapping files (Table 9) and the KEGG database [53] were used to map the output from each tool to gene ontology (GO) molecular functions, EC numbers, and KEGG reactions. A consensus functional annotation was built, and KEGG reactions were included if associated with an EC number in the consensus. Figure 6 shows the workflow followed to construct the consensus functional annotation.

**Table 8:** Tools used in functional annotation of transcripts

Name of tool	References	%Transcripts annotated
KEGG Automatic Annotation Server (KAAS)	[33]	32.6
InterProScan	[34]	71.1
Blast2GO	[35]	56.1
Batch CD-Search against COG database	[36–38]	45.9
Batch CD-Search against PFAM database	[36, 37, 39]	68.6
Batch CD-Search against SMART database	[36, 37, 40]	22.1
Batch CD-Search against TIGR database	[36, 37, 41]	30.3
ScanProsite	[42]	35.8
BLASTP search against BRENDA database (thresholds: 60% identify, E-value 1E-6)	[43, 44]	24.1
HAMAP-Scan	[45]	2.6
GOFEAT (threshold: E-value 1E-5)	[46]	66.9
EFICAz^2.5^	[47]	19.4
TransAAP	[48]	6.8

**Table 9:** Mapping files used in functional annotation of transcripts

Name of mapping file	Source	Date	References
ec2go	http://current.geneontology.org/ontology/external2go/ec2go	2020/06/01	[49]
pfam2go	http://geneontology.org/external2go/pfam2go	2020/04/18	[50]
prosite2go	http://current.geneontology.org/ontology/external2go/prosite2go	2020/04/18	[50]
smart2go	http://current.geneontology.org/ontology/external2go/smart2go	2020/04/18	[50]
hamap2go	http://current.geneontology.org/ontology/external2go/hamap2go	2020/04/18	[51]
rhea2kegg_reaction.tsv	https://ftp.expasy.org/databases/rhea/tsv/rhea2kegg_reaction.tsv	2020/07/10	[52]
TIGRFAMS_GO_LINK	ftp://ftp.jcvi.org/pub/data/TIGRFAMs/	2014/09/17	[41]
TIGRFAMs Complete Listing	http://tigrfams.jcvi.org/cgi-bin/Listing.cgi	2014/09/17	[41]
ko2cog.xl	https://www.genome.jp/kegg/files/ko2cog.xl	2020/07/02	[53]
ko2go.xl	https://www.genome.jp/kegg/files/ko2go.xl	2020/07/02	[53]
ko2tc.xl	https://www.genome.jp/kegg/files/ko2tc.xl	2020/07/10	[53]

### Mapping transcript expression to a reaction-level

The transcript expression data were mapped to a reaction-level following the transcript–reaction associations in iDU1327 and according to the following rules: In the case of an OR relationship, the expression of associated transcripts was summed. In the case of an AND relationship, the minimum expression of associated transcripts was taken. Reactions with expression below the cut-off (TPM < 1) were switched off.

### Transcriptome-guided in silico evolution of constrained citric production

The in silico evolution of constrained citric production was performed using an evolutionary algorithm implemented in Java [19] with changes made to the fitness function and mutation operator as detailed below:

#### (i) Fitness function

The fitness was calculated with respect to T2 by a least-squares fitting procedure

1 $$F = - log_{10} \sum \left( {f_{t} - f_{a} } \right)^{2} ,$$

where *F* is the fitness, *f*_*t*_ is the target flux, and *f*_*a*_ is the actual flux.

The fluxes included were those for five exchange reactions (internal phosphate, glucose, xylose, biomass, and citric acid). The target citric output flux was set to 0.12 in line with in vivo data, and the other target fluxes were set to their original values.

#### (ii) Mutation operator

Flux bounds were subjected to change by the mutation operator, informed by differential expression events (fold change ≥ 2) at both T1 to T2 and T2 to T3, and resulted in flux either being constrained (down-regulation), forced (up-regulation), or unchanged (no differential expression). Mutations were permitted to alter flux bounds within the multiplicative bounds determined by the fold change in expression and fluxes at T1 and T3, with the effect of imposing a limit on the extent of flux constraint and a minimum value for forcing flux. Mutations were not allowed to force flux on reactions without a clear direction of flux or beyond the maximum allowable flux. For initial mutations, the flux bound was set randomly between the minimum flux forced and the maximum allowable flux if forcing flux or between the original flux and the limit of flux constraint if constraining flux. For subsequent mutations, the flux bound was mutated from the existing mutated flux bound. Mutations constraining flux were applied to both lower and upper bounds for reversible reactions. Mutations were performed by adding a small value to the flux bound determined by the double Laplace function. The location parameter, $\mu$, was set to zero, and the scale parameter, $b$, was set according to

2 $$\begin{gathered} b = 0.01\left| B \right|,\,\left| B \right| > 0 \hfill \\ b = 0.001,\,\left| B \right| = 0, \hfill \\ \end{gathered}$$

where $b$ is the scale parameter, and $B$ is the flux bound that the mutation is applied to.

#### (iii) Driving evolution of multiple solutions

A fitness threshold of 6 was applied to identify evolved solutions, and these were analysed to identify their key reactions. This threshold was chosen as at this fitness value the fluxes of selected exchange reactions are sufficiently close to their target values. Each mutated flux bound was evaluated for its contribution to the fitness by complementation with the original flux bound, and the reaction corresponding to the mutation with the greatest contribution to fitness was identified as the key reaction. Flux bounds of the key reaction were then reset to the original across the population and blocked from mutating again. The in silico evolution was run for 150,000 generations, a duration sufficient to allow for the evolution of multiple solutions.

### Processing solutions from in silico evolution to suggest targets for strain optimisation

Solutions from in silico evolution were subjected to optimisation and simplification. Mutated flux bounds were evaluated for their contribution to the solution fitness by resetting them to the original flux bounds, and the mutations were removed if the solution fitness remained over a threshold of 6. Additionally, the mutations on a reaction’s flux bounds were optimised by making small adjustments. Processed solutions were analysed to rank their mutations by contribution to the solution fitness, by individually complementing the mutated flux bounds on each reaction with the original flux bounds. Key reactions were identified by proceeding down the ranked list and successively removing mutations until citric acid output flux increased close to its original unconstrained value. For this purpose, a revised fitness was computed by substituting the target citric acid output flux of 0.12 with the original unconstrained value, and a threshold of 3.5 was applied for the revised fitness to identify the set of key reactions that when returned to their original flux bounds result in optimal citric production. This threshold was chosen as at this fitness value the fluxes of selected exchange reactions are sufficiently close to values that reflect optimal citric production. A set of key reactions was obtained from each processed solution, and from these target suggestions were sourced. A threshold of 50% increase in citric acid output flux at T2 was applied to identify prominent suggested targets that have significant effect in silico.

## Supplementary Information

**Additional file 1:** The iDU1327 model presented as a spreadsheet.

## Data Availability

The datasets used and/or analysed during the current study are available from the corresponding author on reasonable request.
